# Phenomic selection in slash pine multi-temporally using UAV-multispectral imagery

**DOI:** 10.3389/fpls.2023.1156430

**Published:** 2023-08-21

**Authors:** Yanjie Li, Xinyu Yang, Long Tong, Lingling Wang, Liang Xue, Qifu Luan, Jingmin Jiang

**Affiliations:** ^1^ State Key Laboratory of Tree Genetics and Breeding, Chinese Academy of Forestry, Beijing, China; ^2^ Research Institute of Subtropical Forestry, Chinese Academy of Forestry, Fuyang, Hangzhou, Zhejiang, China; ^3^ Key Laboratory of State Forestry and Grassland Administration on Subtropical Forest Cultivation, Fuyang, Hangzhou, Zhejiang, China; ^4^ Key Laboratory of Tree Breeding of Zhejiang Province, Fuyang, Hangzhou, Zhejiang, China; ^5^ Soybean Research Institute, National Center for Soybean Improvement, Key Laboratory of Biology and Genetic Improvement of Soybean (General, Ministry of Agriculture), State Key Laboratory of Crop Genetics and Germplasm Enhancement, Jiangsu Collaborative Innovation Center for Modern Crop Production, College of Agriculture, Nanjing Agricultural University, Nanjing, China; ^6^ Chongqing Academy of Forestry, Chongqing, China; ^7^ Forestry and Water Conservancy Bureau of Fuyang District in Hangzhou, Hangzhou, China

**Keywords:** phenomic selection, forest phenomics, PBWAS, high throughput, time-series

## Abstract

Genomic selection (GS) is an option for plant domestication that offers high efficiency in improving genetics. However, GS is often not feasible for long-lived tree species with large and complex genomes. In this paper, we investigated UAV multispectral imagery in time series to evaluate genetic variation in tree growth and developed a new predictive approach that is independent of sequencing or pedigrees based on multispectral imagery plus vegetation indices (VIs) for slash pine. Results show that temporal factors have a strong influence on the *h^2^* of tree growth traits. High genetic correlations were found in most months, and genetic gain also showed a slight influence on the time series. Using a consistent ranking of family breeding values, optimal slash pine families were selected, obtaining a promising and reliable predictive ability based on multispectral+VIs (MV) alone or on the combination of pedigree and MV. The highest predictive value, ranging from 0.52 to 0.56, was found in July. The methods described in this paper provide new approaches for phenotypic selection (PS) using high-throughput multispectral unmanned aerial vehicle (UAV) technology, which could potentially be used to reduce the generation time for conifer species and increase the genetic granularity independent of sequencing or pedigrees.

## 1 Introduction

Tree breeding primarily mimics the natural selection of breeding domestication based on cycles of selection, mating, and testing that have successfully increased tree productivity and genetically improved tree materials for multiple traits (Pâques, 2013). However, forest trees typically have long breeding cycles and large physical sizes, making breeding and progeny testing complex and expensive (Isik, 2014). Compared to crop breeding, forest tree breeding is still in its infancy (Lyzenga et al., 2021). The development of molecular genetic methods has greatly improved selection efficiency. Many available molecular markers are colocalized with functional genetic variation, and breeders can use these markers to aid breeding (Lande and Thompson, 1990; Wang et al., 2018). The goal of molecular genetics is to identify the polymorphic markers or genes associated with phenotypic variation in target traits (Rasmussen, 2020). However, most target traits are complex and influenced by numerous genes, but each effect is small. Low-throughput marker selection methods, such as microsatellites (Jarne and Lagoda, 1996) and marker-assisted selection (MAS) (Ribaut and Hoisington, 1998), are outdated and not as successful as expected. Therefore, genomic selection (GS) using genome-wide markers has been proposed in breeding (Jannink et al., 2010). GS mainly aims to calculate the genomic estimated breeding value (GEBV) of target traits by estimating the effects of all loci using single nucleotide polymorphism (SNP) markers, resulting in more comprehensive and reliable selection (Newell and Jannink, 2014). GS has been successfully applied in crop breeding, which can greatly improve the prediction of breeding value (BV) and reduce the recurrent cycles of selection. GS is becoming the most popular and successful strategy for predicting breeding values of target traits for selection (Crossa et al., 2017; O’Connor et al., 2021). As high-throughput sequencing becomes more efficient and affordable, interest in GS has increased in forest tree breeding (Grattapaglia et al., 2018; Ukrainetz and Mansfield, 2020). However, GS may not always be appropriate for tree species, especially conifers that have not been whole-genome sequenced, such as slash pine (Scott et al., 2020), because these candidates often have large, uncharacterized, and complex genomes, making rapid assembly of reference genomes difficult; without sufficient funding or prior genome characterization, GS seems out of reach (Rincent et al., 2018).

There are two important kernel functions that have been used primarily in GS, including the Gaussian kernel (GK) and the genomic best linear unbiased predictor (GB) (Cuevas et al., 2016). GB is a linear kernel that uses the marker matrix to compute the genomic relationship matrix, also called the kinship matrix, while GK is a covariance matrix that reveals the complex marker effects and the possible interactions (Cuevas et al., 2019). The prediction of GK usually performs better than GB in a single environmental condition (Bandeira e Sousa et al., 2017). All these kernel functions use a large number of molecular markers to predict the target traits, which is similar to predictive models built using machine or deep learning methods based on near-infrared spectroscopy (NIRS) or hyperspectral data (Yoosefzadeh-Najafabadi et al., 2021; Li et al., 2022). Therefore, it is plausible to use spectral data to estimate the kinship matrix, similar to the use of markers (Van Tassel et al., 2022).

Recently, phenomic selection (PS) has emerged to address these issues by using high-dimensional secondary traits (HDSTs) (e.g., individual sample near-infrared (NIR) spectra or hyperspectral imaging) instead of SNPs to estimate the realized genomic relationship matrix (kinship matrix) between individuals, taking advantage of algorithms and workflows developed for GS (Krause et al., 2019; Adak et al., 2021). PS was first proposed by Rincent et al. (2018), who compared the predictive ability of both NIRS and molecular markers with two types of GS models, including the GB and Bayesian LASSO (BL) models, respectively, and the results showed that using NIRS provided similar or even better predictive results than using molecular markers, depending on the trait of interest and the different types of NIRS. Similar results have been consistently shown in maize and soybean, where the use of NIRS or hyperspectral imaging could generate competitive estimated breeding values, called phenomic estimated breeding values (PEBVs), rather than genomic estimated breeding values (GEBVs) (Adak et al., 2021; Zhu et al., 2021; Weiß et al., 2022). However, phenomic selection using unmanned aerial vehicle (UAV)-based imagery has been less studied.

UAV-based remote sensing has been greatly facilitated for data acquisition by advances in sensor technology, which has the potential to increase fieldwork efficiency with less time to collect spatial information than ground-based spectroscopy and to cover large areas while maintaining accuracy and resolution.

UAVs can acquire various types of data, including spectral, structural, thermal, and feature data, which have been widely used in plant science to estimate various traits (Tsouros et al., 2019). For example, UAV-based multispectral or hyperspectral imagery could be used to estimate leaf chlorophyll content and nitrogen concentration (Zheng et al., 2018), canopy structure information such as height and canopy area from the Light Detection and Ranging (LiDAR) system (Maesano et al., 2020) and real-time kinematic (RTK) positioning system (Tao et al., 2021) for plant biomass (Masjedi and Crawford, 2020; Nik Effendi et al., 2021) and grain yield prediction (Li et al., 2020).

In addition, UAV-based imagery also provides a high-precision, high-throughput method for field-based multitemporal phenotyping data collection in the context of plant breeding. This allows for the provision of dynamic information on plant growth and performance (Díaz-Varela et al., 2015; Song et al., 2022). For example, the height data of sorghum and maize from different groups of breeding material estimated by UAV-based imagery have been used to detect the different growth stages (Han et al., 2018; Pugh et al., 2018). UAV-based thermal imagery has been used for high-throughput field phenotyping of black poplar response to drought (Ludovisi et al., 2017). Therefore, UAV-based imagery is very helpful for forest inventories because traditional measurements of tree height and crown growth are difficult due to the difficulty in determining the top of the tree crown and the two cross-crown diameters to simplify the calculation of crown area (Guerra-Hernández et al., 2017). With the specific wavelengths and the RTK system, UAV-based multispectral imagery allows us to obtain the growth parameters as well as the content of physiological and photosynthetic pigments in the leaves.

Although there are achievements in growth trait detection and leaf physiological prediction for plant breeding based on UAV-based imagery, no research has been found on the use of multitemporal HDSTs to perform phenomic selection of growth traits in slash pine. In previous studies (Tao et al., 2021; Song et al., 2022), we developed a UAV-based multispectral imagery phenotyping method that successfully detected growth parameters such as tree height, crown area, and biomass, which were combined to estimate genetic variation with various vegetation indices (VIs) in slash pine (*Pinus elliottii*). However, previous studies did not consider using the multispectral as an indicator to predict the genetic parameter. Here, we further combined this methodology with multitemporal growth and multispectral data in a slash pine breeding plantation to evaluate the potential of linking high-throughput phenotyping with growth parameters to perform phenomic selection.

Slash pine is a typical conifer with a large, uncharacterized, and complex genome, and the reference genome of slash pine is still unavailable; therefore, genetic studies of slash pine are mainly based on the transcriptome (Diao et al., 2019; Ding et al., 2022).

Therefore, we used slash pine breeding populations as model materials to evaluate a novel approach for low-cost, high-throughput phenomic selection of growth trait-based multispectral images. Our objectives were to 1) estimate genetic variation in growth traits in time series using UAV multispectral imagery; 2) evaluate the predictive ability of the GB and GK models using time series multispectral data for phenomic selection; and 3) develop new predictive selection approaches that are independent of sequencing or pedigrees in trees, especially in conifer breeding programs.

## 2 Methods and materials

### 2.1 Site description

The study was conducted on a slash pine population in a national forest farm in Anhui, China; details can be found in Song et al. (2022). There were twenty open-pollinated families with a lattice incomplete block single-tree plot design planted in 2013 within two sites. Each block contained 20 trees, and the spacing between each tree per block was 2 m×3 m. Each tree represented a single family, with no replications within a block. There were 2 sites, and each site contained 20 blocks. 30% of the trees died (240/800) during these years. In total, there were 560 remaining individual trees. Tree canopies did not overlap. This region has a subtropical temperate climate with an average temperature of 15°C.

### 2.2 UAV flights and field data collection

Flights were performed monthly in 2021 (at the age of 8 years) using DJI Phantom 4 Multispectral (DJI, Shenzhen, China), which has 1 RGB camera and 5 wavelengths (450 nm ± 16 nm, 560 nm ± 16 nm, 650 nm ± 16 nm, 730 nm ± 16 nm, 840 nm ± 16 nm). This UAV is equipped with an RTK system that can reduce the horizontal and vertical positioning errors to 0.03 m and 0.06 m, respectively. The output images from each multispectral camera are in TIF format with a resolution of 1600×1300 pixels.

Flights were conducted at a fixed height of 35 m above ground level during a sunny and less windy day in each month to ensure high accuracy requirements and to reduce any systematic bias caused by environmental factors. A standard reflectance panel was used during each flight to improve the consistency of the spectral data. The operation was set to 80% overlap between images and a forward speed of 5 m/s during the flights. The original images were normalized to adjust the data and align the spectral information across the images. The total area covered was 4.5 ha and the duration of each flight was 1 hour. During the Covid-19, the field trip was strictly restricted in February 2021, so data were not available. To validate the accuracy of tree height and crown area (CA) measurement by UAV images, the ground truth data of tree height and CA were measured by randomly selected 100 trees in July of 2021, with the high accuracy of RTK system, the UAV-based tree height and CA have a high correlation with the ground truth, with the R2 value higher than 0.85 (Song et al., 2022).

### 2.3 Image processing

In this study, the image processing methodology employed a series of steps to extract essential information from the original multispectral images of the plantation. The initial data processing involved the use of DJI Terra software (version 3.3.0, Shenzhen, China) to generate multispectral orthomosaics and dense 3D point clouds of the entire plantation. These orthomosaic images, along with the 3D point clouds, served as the basis for further analysis.

The orthomosaic images and 3D point clouds were then further processed using the R software version 4.2.0 and the *lidR* package version 4.0.0 (Roussel et al., 2020). The first step in data analysis was the classification of ground points within the 3D point clouds using the cloth simulation filtering (CSF) function, as proposed by Zhang et al. (2016).This step was critical for creating a digital terrain model (DTM) that accurately represented the bare ground surface.

With the classified ground points, the next step was to create digital surface models (DSM) using a point-to-grid algorithm. The resolution of both the DTM and the DSM was set at 0.5 m, ensuring a high level of detail in the representation of terrain and surface objects. The difference between the DSM and the DTM provided the canopy height models (CHM), which indicate the height of vegetation above the ground surface.

Using the CHM, individual trees were detected using the *dalponte2016* function with specific criteria, including a minimum height threshold of 2.6 m and a maximum crown diameter of 2.5 m. This step allowed for the identification and delineation of individual trees within the study area.

For each detected individual tree, tree-level attributes such as tree height and crown area were manually labeled. In addition, relevant family, site, and block information was associated with each tree to improve the accuracy and context of the tree-level data.

To represent the spatial extent of individual tree canopies, tree crown polygons were generated from the manually labeled crown areas using the raster package (Hijmans et al., 2015). Finally, the spectra of each individual tree were extracted using the tree crown polygons. This extraction involved collecting spectral information from the multispectral orthomosaic images for all pixels within the boundary of each tree crown.

Similar to previous studies (Tao et al., 2021; Song et al., 2022), fifteen vegetation indices (VIs) were calculated for each pixel from all extracted tree images (**Table 1**). These VIs were then averaged at the tree level based on the red, green, blue, red edge, and near-infrared (NIR) spectra, providing valuable insights into the vegetation health and other biophysical characteristics of the individual trees. The comprehensive image processing methodology described above ensured accurate data extraction and analysis, allowing researchers to gain valuable information about the structure, health, and vegetation dynamics of the plantation.

**Table 1:** The spectral indices used in this study. $\lambda_{r}$ , $\lambda_{b}\;$ and $\;\lambda_{g}$ are the reflectances at wavelength λ .

Name	Abbrev.	Equation	Reference
Normalized difference vegetation index	NDVI	(NIR−R)/(NIR+R)	Peñuelas et al. (1993)
Optimized soil adjusted vegetation index	OSAVI	((NIR−R)(1+0.16))/((NIR+R+0.16))	Rondeaux et al. (1996)
Green normalized difference vegetation index	GNDVI	(NIR−G)/(NIR+G)	Gitelson et al. (1996)
Soil adjusted vegetation index	SAVI	((NIR−R)(1+0.5))/((NIR+R+0.5))	Huete (1988)
Modified soil adjusted vegetation index	MSAVI	(2NIR+1−√((2NIR+1)^2−8(NIR−R) ))/2	Qi et al. (1994)
Triangular greenness index	TGI	$-0.5\left[\left(\lambda_{r}-\lambda_{b}\right)\left(R-G\right)-\left(\lambda_{r}-\lambda_{g}\right)\left(R-B\right)\right]$	(Hunt et al., 2011)
Green leaf index	GLI	(2G−R−B)/(2G+R+B)	(Louhaichi et al., 2001)
Triangular vegetation index	TVI	0.5[120(N-G)-200(R-G)]	(Broge and Leblanc, 2001)
Red edge chlorophyll index	RECI	NIR/E−1	Gitelson et al. (2003)
Leaf chlorophyll index	LCI	(NIR−E)/(NIR+R)	Pu et al. (2008)
Anthocyanin reflectance index	ARI	G/NIR	van den Berg and Perkins (2005)
Modified green red vegetation index	MGRVI	(G^2−R^2)/(G^2+R^2 )	Bendig et al. (2015)
Modified anthocyanin reflectance index	MARI	(G^(−1)−E^(−1))/NIR	Gitelson et al. (2006)
Normalized difference red edge index	NDRE	(NIR−E)/(NIR+E)	Barnes et al. (2000)
Red green blue vegetation index	RGBVI	(G^2−R×B)/(G^2+R×B)	Bendig et al. (2015)

R: red bands, G: green bands, B: blue bands, E: red edge bands, NIR: near infrared bands.

### 2.4 Genetic parameters

Estimates of genetic parameters for slash pine growth traits in each month of the year were collected by fitting a general multiple mixed linear model using restricted maximum likelihood (REML); details can be found in (Li et al., 2018; Cuevas et al., 2019). A brief description can be expressed as:


(1) $$y_{i}\;=x_{i}m+b_{i}+f_{i}+e_{i}$$

$y_{i}$ is a vector containing the phenotypic values for both traits (tree height and crown area) for the individual. $x_{i}$ is a vector linking the fixed effects m to the observations for the individual. m is a vector of fixed effect coefficients for the traits. $b_{i}$ is a vector representing the random block effects for the individual. $f_{i}$ is a vector representing the random family effects for the individual. $e_{i}$ is a vector representing the random residual effects for the individual. By stacking these vectors for all trees, we can represent the overall model equation as:


(2) $$Y\;=Xm+Z_{1}b+Z_{2}f+e$$

where Y is a vector of phenotypic observations (containing measurements for both traits). m is a vector of fixed effects, representing the overall mean. b , f , and e are vectors of bivariate random effects for block, family, and residual effects, respectively.X is the incidence matrix linking observations to the fixed effects. $Z_{1}$ and $Z_{2}$ are incidence matrices linking observations to the appropriate random effects for block and family, respectively. In this model, the fixed effects represented by m (overall mean) are connected to the phenotypic observations through the incidence matrix X. Similarly, the random effects for block and family, represented by b and f respectively, are linked to the observations through the incidence matrices $Z_{1}$ and $Z_{2}$ . The vector e accounts for the residual effects, which are not explained by the fixed or random effects. For each month, the equation can be:


(3) $$Y_{i}\;=X_{i}m+Z_{i_{1}}b_{i}+Z_{i_{2}}f_{i}+e_{i}$$

Where $Y_{i}$ is the vector of bivariate phenotypic observations for the $i_{th}$ month. m is the vector of fixed effects, representing the overall mean. $b_{i}$ , $f_{i}$ , and $e_{i}$ are the vectors of bivariate random effects for block, family, and residual effects, respectively, specific to the $i_{th}$ month. $X_{i}$ is the incidence matrix linking observations to the fixed effects for the ith month. $Z_{i}1$ and $Z_{i}2$ are the incidence matrices linking observations to the appropriate random effects for block and family, respectively, for the $i_{th}$ month. The variance components were used to estimate the temporal narrow sense of *h^2^* for trait i  and the genetic correlations ( $r_{g_{ij}}$ ) between trait i and trait j ,


(2) $$h_{i}^{2}=\frac{2.5\sigma_{fi}^{2}}{\sigma_{fi}^{2}+\sigma_{bi}^{2}+\sigma_{ei}^{2}}$$

(3) $$r_{g_{ij}=}\frac{\sigma_{f_{ij}}^{\;}}{\sqrt{\sigma_{f_{i}\;}^{2}+\sigma_{f_{j}\;}^{2}}}$$

where $\sigma_{fi}^{2}$ , $\sigma_{bi}^{2}\;$ and $\;\sigma_{ei}^{2}\;$ are the temporal family, block and residual variance for trait i , respectively, and $\sigma_{f_{ij}}^{\;}$ is the estimated family covariance between trait i and trait j . The genetic gain represents the effectiveness of tree improvement and is measured by the change in the mean breeding value of each trait population. In this study, genetic gain ( $\text{Δ}G_{R}$ ) of each trait for each month was calculated by subtracting the mean breeding value of selected ratio growth traits from the total mean of growth traits by breeding value.


$$\text{Δ}G_{R}=M_{BV}\times r\;-T_{BV}$$

Where $T_{BV}$ is the total mean of the growth traits determined by breeding value, $M_{BV}$ is the mean breeding value of the top selected proportions ( r ) of the growth traits in descending order. The variable r denotes the proportion of growth traits selected as top performers.

### 2.5 Kernel methods

We performed two important GS methods, including GB and GK kernels (Cuevas et al., 2019), to compare the phenomic prediction accuracy, and we used multispectral as input instead of SNP data. GB is a standard linear kernel, usually referred to as the genomic relationship matrix (Cuevas et al., 2016). GB is described as $GB=\frac{XX^{\prime }}{p}$ , where X in our study is the kernel matrix formed based on the multispectral and VIs matrix (M BLUP). GK, defined as $GK=\text{exp}\left(-hd_{ii}^{2}/q\right)$ , is different from GB, which is defined as the semiparametric model reproducing kernel Hilbert spaces (RKHS) and appears as a reproducing kernel(González-Camacho et al., 2012), where *q* and *h* are the median of the Euclidean squared distance and the bandwidth parameter affecting the covariance decay rate between genotypes, respectively. Specifically, for each month, we randomly divided the data into an 80% training set for model training and a 20% validation set for model validation. To evaluate model stability, the data were randomly divided 100 times for model training.

### 2.6 Phenotyping-based Wide Association Analysis (PBWAS)

The PBWAS in our study was conducted according to the principles of GWAS (Genome-Wide Association Study) methodology. We considered each temporal month as a chromosome, and a genome-wide association analysis (GWAS) was applied to detect the multispectral and VIs (20 variables) related to the growth traits at each temporal month level. Thresholds of P< 10^-3 were used as the significance level to identify associations between variables and traits. While a typical threshold for GWAS is usually around 10^-4 to 10^-5, we chose a relatively lower threshold given the smaller size and scope of our study compared to traditional GWAS studies with larger genomic datasets. The lower threshold allowed us to identify potentially meaningful associations between the multispectral and VIs traits and tree growth traits in the context of our specific study using spectral data.

All statistical analyses were performed in R software. The *BGGE* package (Granato et al., 2018) was used for GB and GK model calibration, the *sommer* package was used for genetic parameter analysis (Covarrubias-Pazaran, 2016), the *statgenGWAS* package (van Rossum and Kruijer, 2020) was used for PBWAS analysis, and the *ggtree* (Yu et al., 2017; Yu, 2020), *ggplot2* (Wickham, 2011) and *CMplot* packages (LiLin-Yin, 2022) were used for data visualization.

## 3 Results

### 3.1 The growth of height and CA in different months

**Figure 1:**
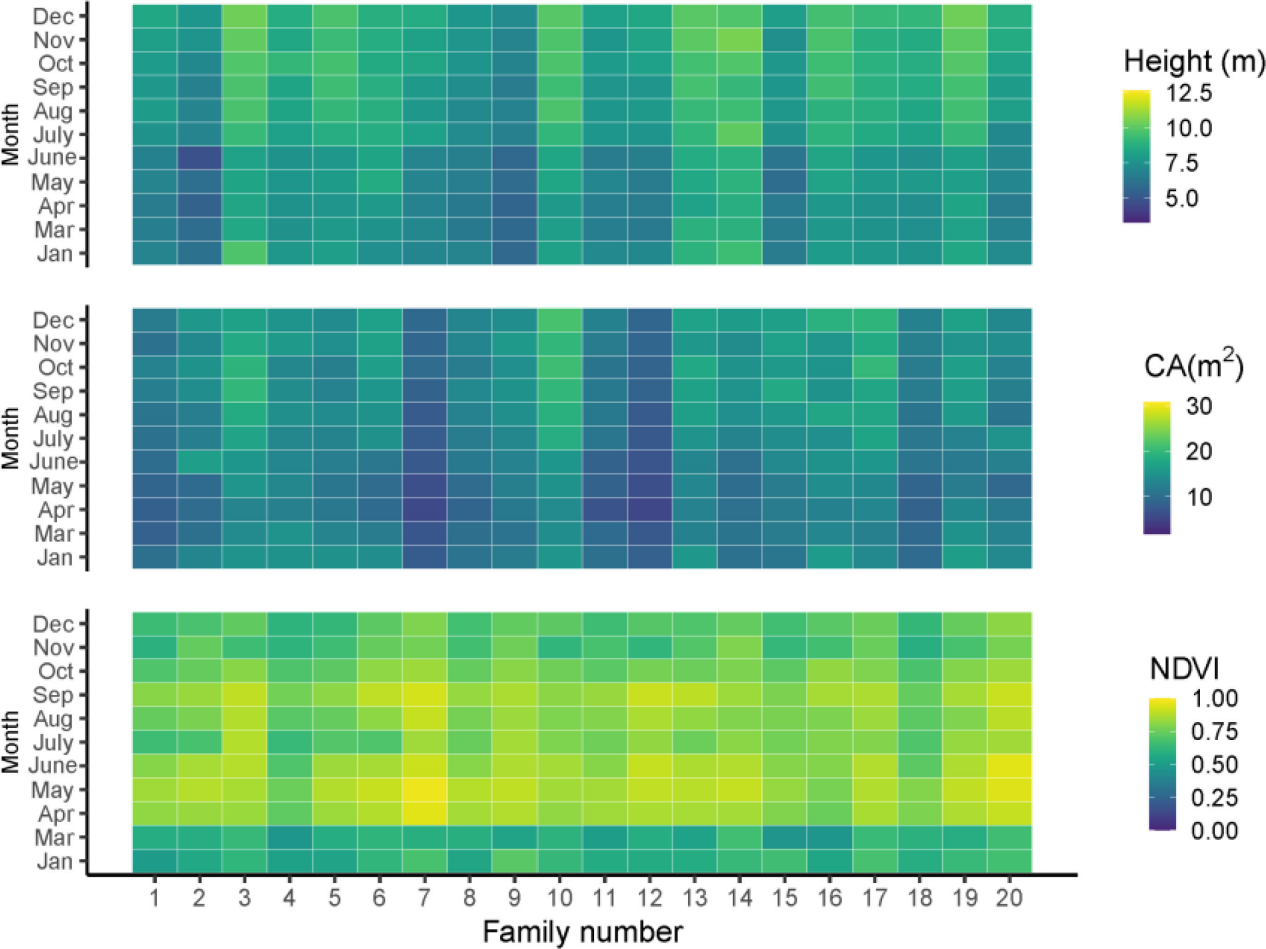
The growth traits of tree height, CA and NDVI in different months. Jan, Mar, Apr, May, June, July, Aug, Sep, Oct, Nov, Dec are represented as January, March, April, May, June, July, August, September, November, December, respectively.

**Figure 2:**
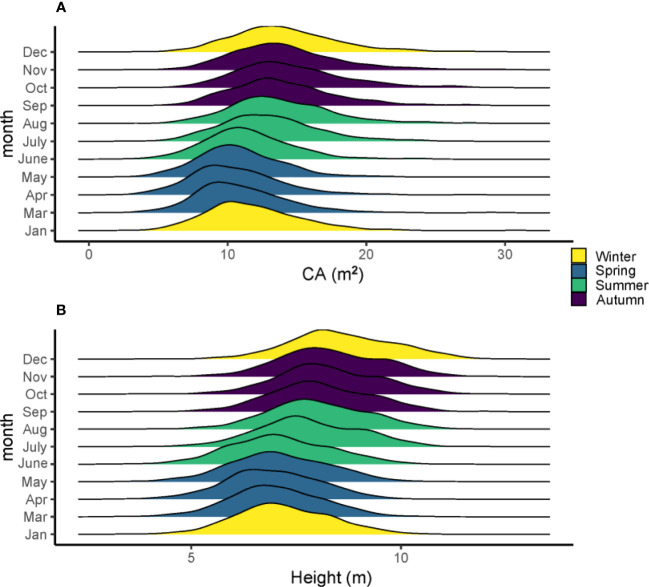
The density mean of tree height **(A)** and CA **(B)** in different months and seasons of the 560 slash pines in 2021.

The average growth trait performance of 20 families is shown in **Figure 1**. Since the growth rate can reflect the percentage change in the indicator over a given time horizon, it can be seen that the growth rate varies considerably among the families during the one-year growth period according to **Figure 1**. The NDVI shows that all trees have a high growth rate from April to September and a slow growth rate from December to March, and families 3, 5, 10, 13, 14, 16, and 19 have a higher mean tree height than other families. However, not all families had high mean tree height followed by high mean CA; only three families, including 3, 10, 16, had both high tree height and CA. Families 7 and 12 had relatively lower mean tree height and CA than the other families, but their growth rate (NDVI) from April to September was high. In general, the total amount of tree height and CA started to increase in summer and slowed down in winter (**Figures 2A, B**).

### 3.2 Genetic variation, correlations and family selection

**Figure 3:**
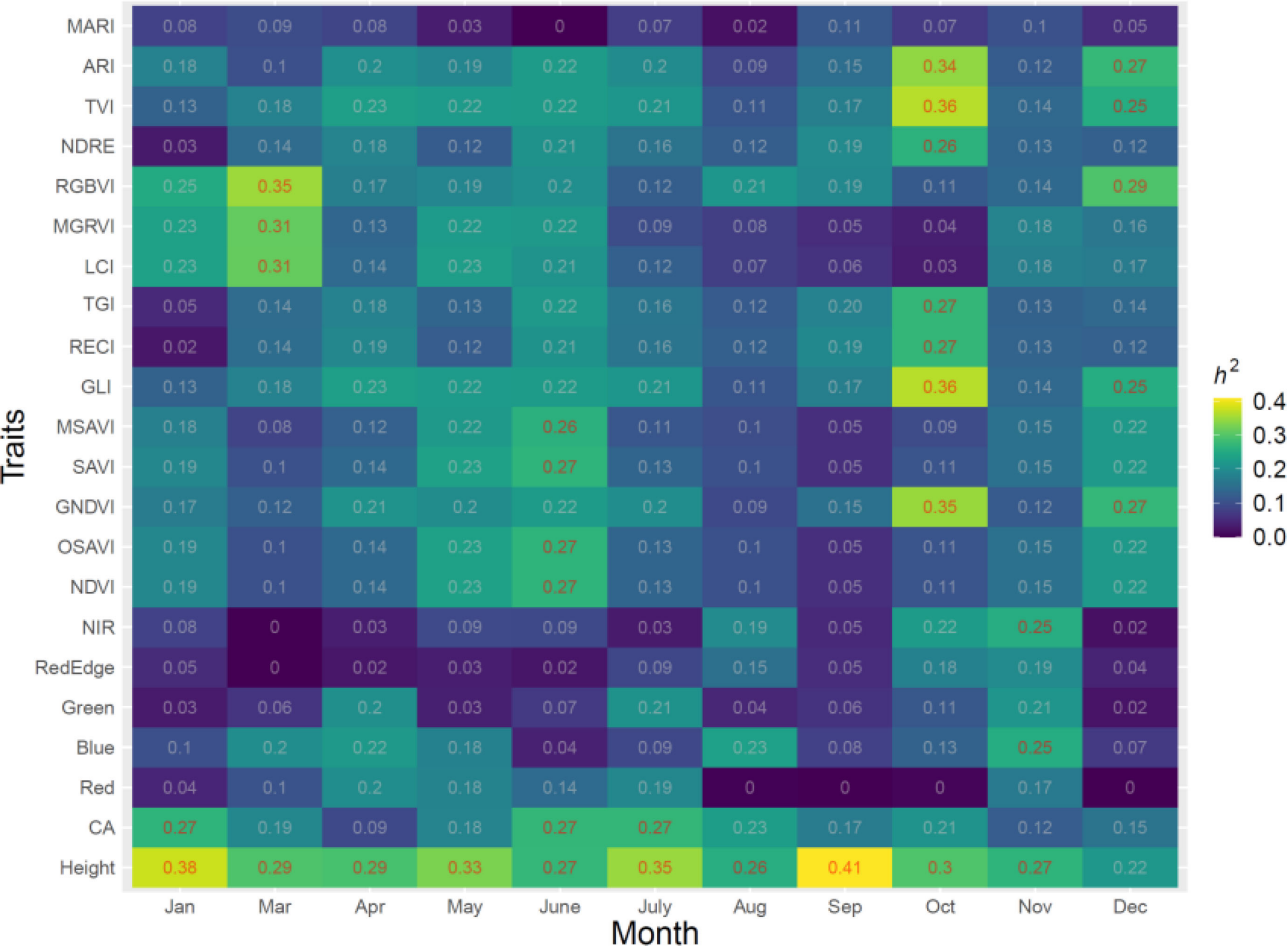
Estimates of *h^2^* from 11 months in 2021 for all traits, including five spectral bands, vegetation indices (VIs), and growth traits.

The variation of the estimate *h^2^* for tree height, CA, VIs and the spectral bands over 11 months is shown in **Figure 3**. A range of *h^2^* from 0 to 0.41 was found for all traits. Temporal phenotypes have a strong influence on the *h^2^* estimates for all traits. All spectral bands including red, blue, green and NIR had relatively low *h^2^* in all months with a range of 0 to 0.25. RGBVI, MGRVI, and LCI had moderate *h^2^* in March, with *h^2^* values of 0.35, 0.31, and 0.31, respectively, but low *h^2^* values in all other months. The *h^2^* values of ARI, MACI, NDRE, GCI, GNDVI, LCI and RECI in the month of October also showed relatively high values compared to other months, with a range of 0.26 to 0.36. Tree height showed a strong stable *h^2^* in all months except Dec, ranging from 0.26 to 0.41. The highest *h^2^* for tree height was found in September, with a value of 0.41, but all spectral and VIs in September were low, with a range from 0 to 0.19. The months had a strong influence on the *h^2^* of CA; the highest *h^2^* of CA was found in January, June and July, and the lowest *h^2^* was found in April, with a value of 0.09.

**Figure 4:**
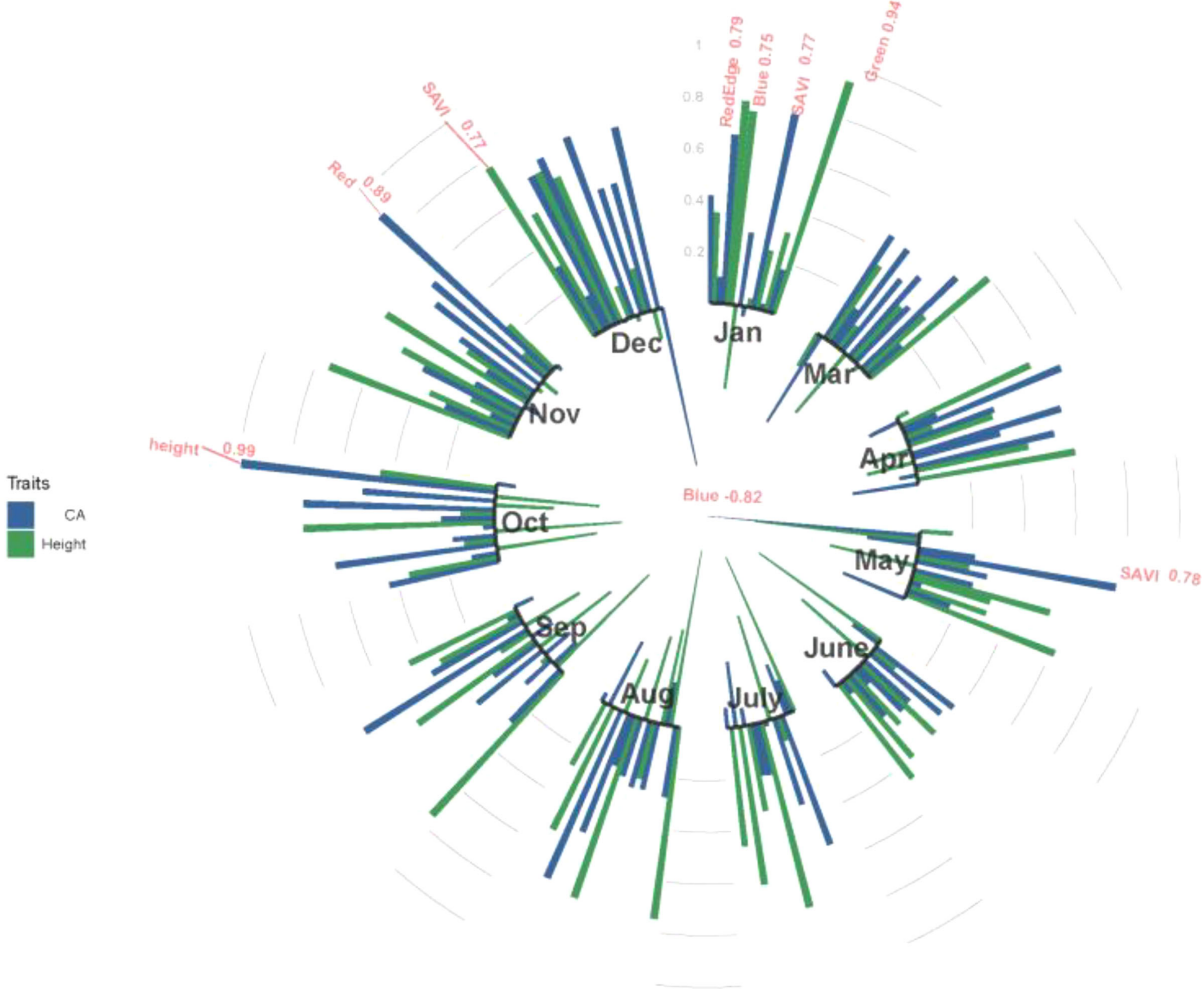
The genetic correlations between tree height and CA and multispectral and VIs at 11 months in 2021. Red indicates genetic correlations above 0.75 in absolute value. Blue color indicates CA; green color indicates height.

**Figure 4** shows the estimated genetic correlations between multispectral, VIs and tree growth traits (height and CA) in different months. High genetic correlations were found from January and July to December. Multispectral and VIs have no significant genetic correlation with tree height or CA in March, April and June, and the highest genetic correlations between tree height and CA were found in October, with an rg value of 0.99. A large number of correlations between multispectral, VIs and tree growth traits (height and CA) were found in January, and red edge, blue and green spectra had a significant positive correlation with CA, with rg values of 0.79, 0.75 and 0.94, respectively. SAVI has a significant positive correlation with height in December (rg=0.77) and CA in May (rg=0.78), and the red spectra also have a strong positive correlation with CA (rg=0.89). In addition, a strong negative correlation was found between the blue spectra and CA in May (rg=-0.82).

**Figure 5:**
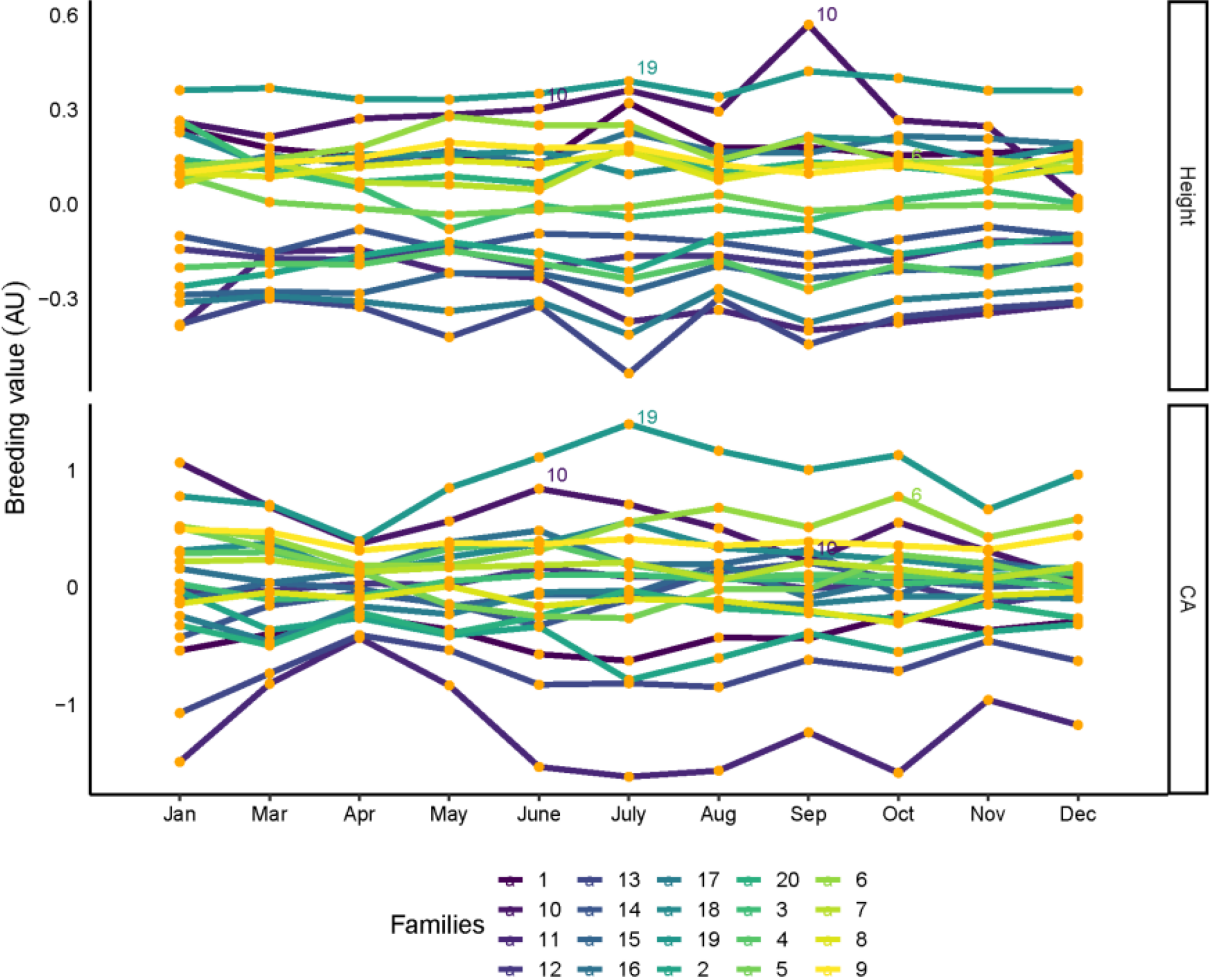
Family rankings for tree height and CA in slash pine in different months. Each line represent one family. Family values are expressed as deviation from each trait mean. AU: arbitrary units.

The breeding values ranked between multitemporal of all families for tree height, CA, are shown in **Figure 5**. Although each family has variable breeding values in different months, most of the families are consistent between each month, and breeding selection is possible for high tree height and CA families in certain months. For tree height, family 19 had the highest breeding values, in addition to families 6 and 10, which also had the highest breeding values in most months. For CA, families 6, 10 and 19 were found to have the highest breeding values for month influence. Family 19 was also selected in January, March, May, November, and December. Families 6, 10 and 19 show the highest breeding value for tree height and CA.

### 3.3 Genetic gain

**Figure 6:**
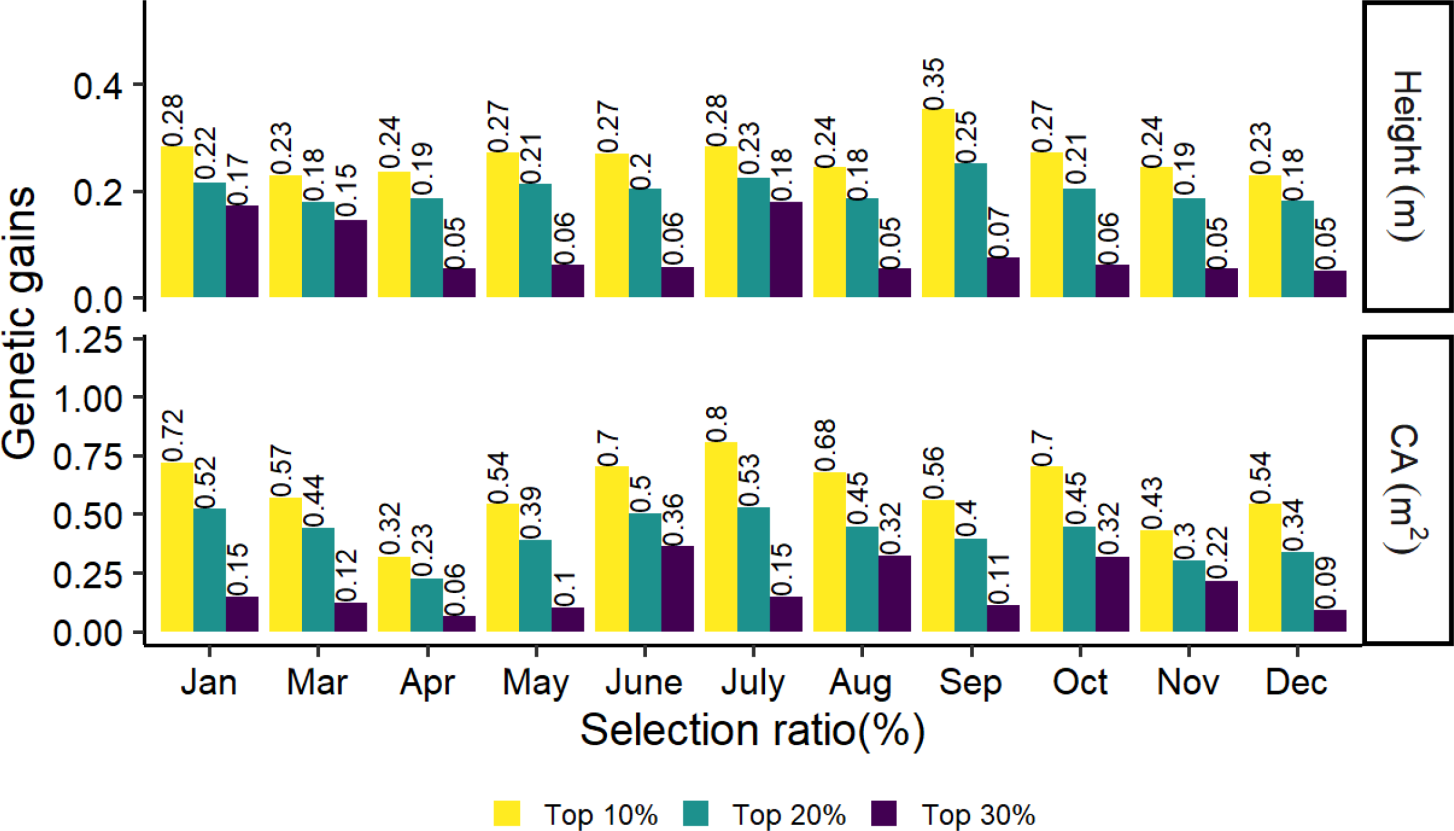
Realized genetic gains of tree height and CA traits at age 8 for slash pine at different months in 2021.

The top 10%, 20% and 30% genetic gains of the families for tree height and crown in different months are shown in **Figure 6**. The highest and lowest genetic gains for tree height and CA with strong selection rates (top 10% and 20%) were found in September, July, and December, April, with values of 0.35, 0.25, 0.8, and 0.53 for the highest and 0.23, 0.18, 0.32, and 0.23 for the lowest, respectively. In general, genetic gains increased as stronger selection rates were applied to tree height and CA.

### 3.4 Phenomic selection using the GB and GK kernels

**Figure 7:**
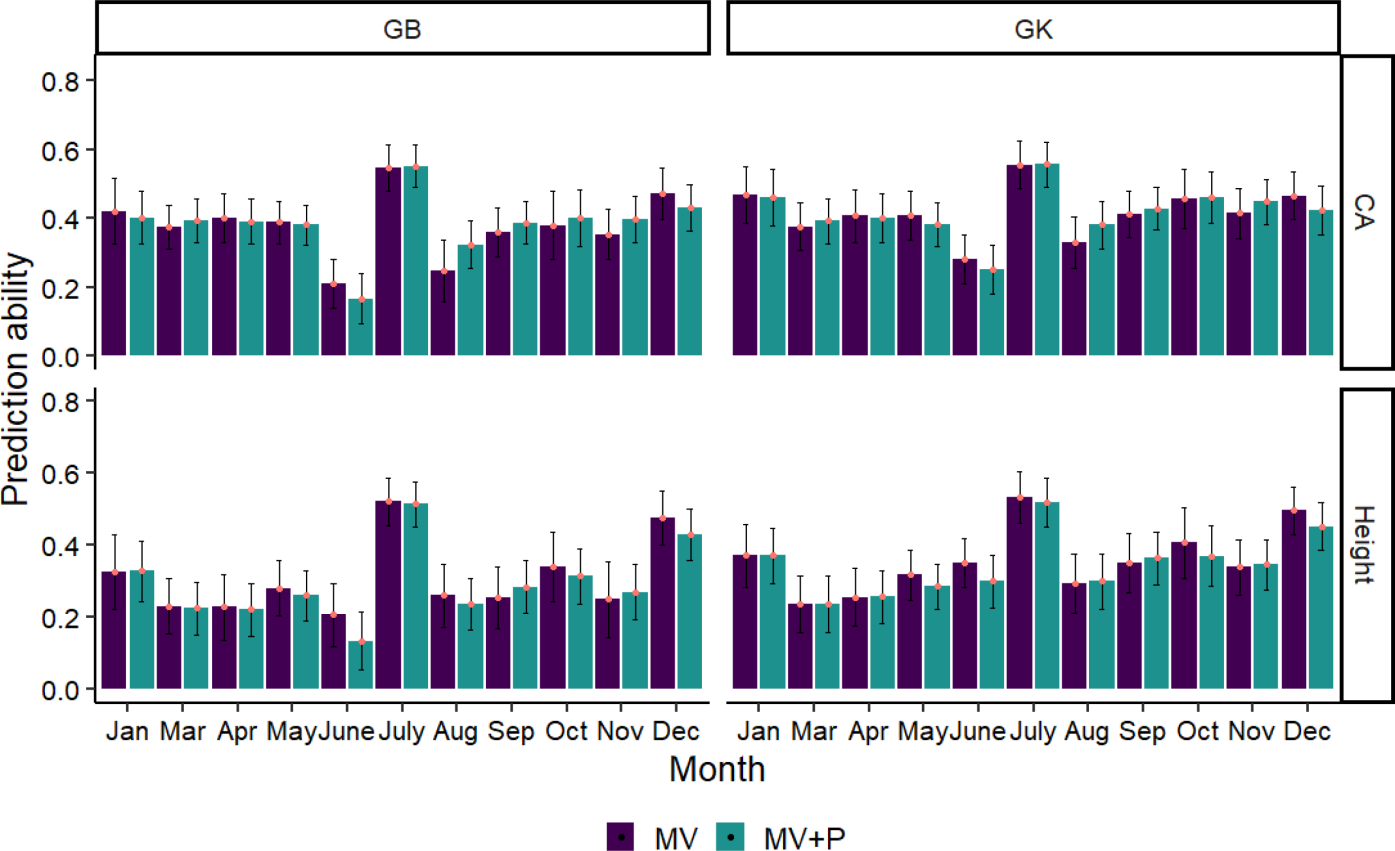
Average Pearson’s correlations between observed and predicted values with standard deviation for 2 methods with 80% of the families in the training set and 20% of the families in the test set. Methods GB and GK are GBLUP and Gaussian Kernel, respectively. The black line in each bar represents the standard deviation (SD). The SD was calculated by training the model on 100 randomly divided subsets of the data and obtaining the standard deviation of the predicted values across these subsets.

The phenomic selection based only on multispectral+VIs (MV) and the combination of MV and pedigree (MV+P) using the GB and GK models is shown in **Figure 7**. Temporal time influenced the predictive ability of PS, with a range from 0.13 to 0.56 for all traits using two kernels (GB, GK). The average prediction of GB is similar to that of the nonlinear kernel GK in all cases. Pedigree does not improve the prediction ability compared to the kernels using MV+P. Interestingly, the combination of pedigree with MV shows similar prediction accuracy compared to the prediction using MV only for the two kernels in some months (December, October, June, May), but similar in January, March, and April. The highest prediction ability for tree height and CA using GB and GK was found in July, with a prediction ability value ranging from 0.52 to 0.56, followed by Dec. The lowest prediction ability for tree height and CA was found in June using GB and March and June using GK, respectively.

### 3.5 PBWAS

**Figure 8:**
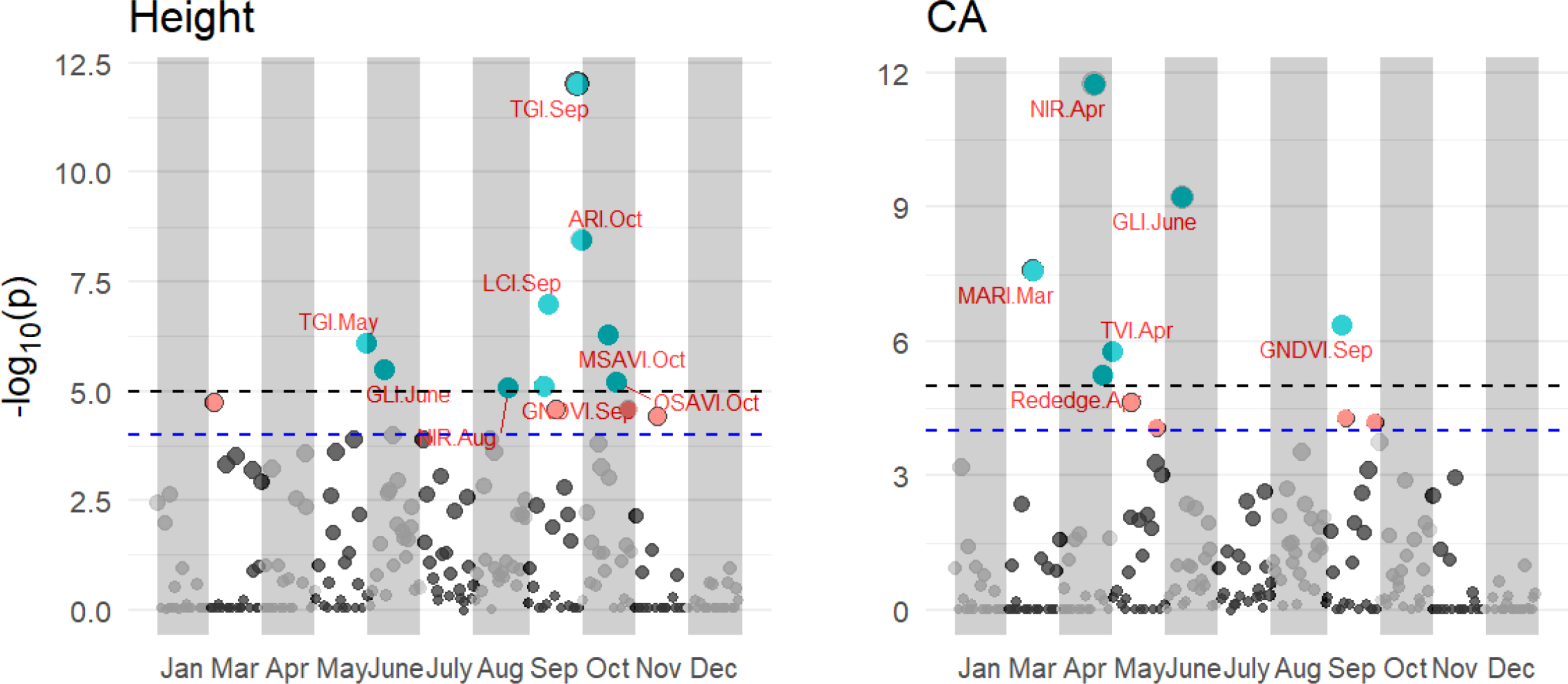
Results of the PBWAS using GWAS methodology based on multispectral and VIs for tree height and CA in 11 months in 2021. Each dot in a different month is a representation of that multispectral or VIs.

PBWAS reveals 15 associations between significant multispectral, VIs and tree height and CA with P< 10^-3^ in these 11 months (**Figure 8**). Tree height was associated with 9 VIs from Jan to Dec, including TGI in May and Sep, GLI in June, NIR in Aug, GNDVI in Sep and ARI, MSAVI, and OSAVI in Oct. Six significant associations emerged between multispectral, VIs and tree CA, including MARI in Mar, Rededge and NIR in Apr, GLI in June and GNDVI in Sep. Among those, the GLI in June and GNDVI in Sep were associated with both tree height and CA. Time series significantly influence the association between multispectral, VIs and tree growth traits. No associations were found to emerge with tree height in Jan, Mar, Apr, July, Nov and Dec and Jan, May, July, Aug, Oct, Nov and Dec for tree CA.

## 4 Discussion

UAV-based imagery has been shown to predict tree growth traits at high throughput and to be used for breeding selection in various tree species (Ota et al., 2017; Jang et al., 2020; Jones et al., 2020; Rallo et al., 2020). Equipped with the RTK system, UAV multispectral imagery provided high accuracy of 3D point cloud data and spectral data for individual trees in forest plantations (Tao et al., 2021). Supportive results were reported by Volpato et al. (2021), who found that postprocessed kinematic (PPK) corrections are an affordable method for plant height, and that PPK or RTK corrections could greatly increase the accuracy of image georeferencing and provide a promising method for plant height. Therefore, UAV imagery is well suited to monitor plant growth traits in a long time series, and successful studies have been conducted in agriculture to estimate growth and yield for breeding selection purposes, including soybean (Borra-Serrano et al., 2020), cotton (Ashapure et al., 2020), sorghum (Masjedi et al., 2020) and tomato (Chang et al., 2021). However, there is limited research on monitoring tree growth in time series for breeding selection purposes (Guerra-Hernández et al., 2017; Solvin et al., 2020). Our study is the first to apply UAVs for tree growth trait identification and the use of multispectral data to perform multitemporal phenomic selection for tree growth traits in a slash pine breeding plantation. This comprehensive approach integrating UAV, multispectral data and multi-temporal analysis represents a unique contribution to the field of tree growth trait identification and phenomic selection in a breeding program. UAV imagery provides a cost- and time-saving phenotyping method for individual tree estimation of growth-related traits, greatly improving data collection over different months or years and characterization of the genetic basis underlying phenotypic differentiation (Masjedi and Crawford, 2020). Our approach collected growth information through multitemporal flights (n=11), each with low computational time, using a low-cost UAV device. This approach has been shown to provide accurate estimates of growth characteristics and VIs in slash pine plantations (Tao et al., 2021). Similar indices have been widely used in many studies at the individual tree level (Santini et al., 2019b; Santini et al., 2021).

Tree height and CA differences were detected among families and increased during spring (March, April, May) and summer (June, July and August) with the increase of tree growth characteristics. However, with the limitation of low quality of RGB camera, there are some tree height and CA do not extract correctly. which occurred that the tree height and CA for some trees from January to May have a trend of decreasing. The *h^2^* of tree height remained relatively stable during the whole growth year in 2021, with a range from 0.22 to 0.41, and tree CA did not have a stable *h^2^*. The highest *h^2^* for CA was found in June and July. These results are consistent with our previous study in which tree height and CA had moderate *h^2^* values of 0.37 and 0.30, respectively, in July (Zhaoying Song et al., 2022). Moderate genetic variability in tree growth traits has also been found in other tree species, for example, a range of 0.21 to 0.30 and 0.19 to 0.28 *h^2^* for tree height in different ages of Norway spruce (*Picea abies* L. Karst.) were found by Solvin et al. (2020) using UAV imagery. The consistency of the families ranked in different months, and the moderate *h^2^*, selected families with high genetic gains for both tree height and CA at different selection ratios. In addition, the best month of selection for tree height and CA was also found in our study.

Most multispectral and VIs in this paper have a large positive or negative genetic correlation with tree height and CA in different months, and multispectral and VIs have been shown to have a strong correlation with plant photosynthetic status, which has the potential to be used in plant phenomics approaches. Santini et al. (2021) proposed that VIs show a strong relationship with aboveground growth traits, whereas leaf biochemistry has no significant effect on tree growth (Santini et al., 2019a). The strong genetic correlation between VIs and tree growth traits suggests that a PS based on these factors is possible. In this paper, we aim to apply a PS approach similar to that first reported by Rincent et al. (2018), who used NIRS as a low-cost, high-throughput phenotype to make predictions instead of genetic markers. The only difference in our study is that we use five spectral bands and many VIs as inputs instead of markers to perform PS. Most of the canopy spectrum and VIs showed genetic variability in different months, which is consistent with the results of Rincent et al. (2018), who found that VIS-NIR wavelengths between 400-2500 nm mostly showed genetic variability. Therefore, Vis, like NIRS, should be used to process PS instead of genetic markers. We collected the spectrum and VIs from different growth months to determine the temporal influence on PS performance. Since we do not have marker data, the GB and GK models were performed based on MV and the combination of MV and pedigree data. Although the growth time influences the PS prediction, we still obtained the highest PS prediction ability in July with a range of 0.52 to 0.56. A supported study was reported by Rincent et al. (2018), who used NIRs and obtained moderate PS predictive abilities ranging from 0.34 to 0.53 for wood properties in black poplar, but slightly lower predictive abilities than those with SNPs. There were no significant differences in the accuracy of the PS models generated by GB and GK in our study. Moreover, the accuracy of both models was higher than the range reported by Cuevas et al. (2019), for wheat data, where GB and GK yielded a range between 0.349 and 0.367 for grain yield prediction using NIR spectroscopy. The PS models generated in our study also outperformed models using only genomic markers or a combination of genomic markers, pedigrees, and NIRS, which had predictive abilities ranging from 0.40 to 0.47. Cuevas et al. (2019) also reported that the markers obtained slightly higher correlations between observed and predicted values than pedigree + NIRS, indicating that even if PS is less accurate than GS in some cases, it could be a feasible alternative and reliable method for filtering the poor performing germplasm when markers are not available, which could be a low-cost and high-throughput method independent of sequencing or pedigrees for tree breeding selection, especially for tree species with large and complex genomes without prior genome characterization, GS is often costly and inaccessible.

We used GWAS methodology to reveal the significant VIs and spectra associated with growth traits at different growth times, which we call PBWAS. The results demonstrated the effectiveness of combining phenomic information with UAV imagery to characterize growth differentiation at different growth times in slash pine. We identified relevant VI phenotypic associations for tree height and CA in several months. These associations were inconsistent across months for tree height and CA, as reported by Roberts et al. (2016). NDVI, OSAVI, and GNDVI were found to be saturated at high leaf area and may not capture individual differences in tree growth. These indices were also found in our work to be highly associated with tree growth in different months. The strongest correlations between VIs and tree growth traits were TGI and LCI in Sep, ARI in Oct, GLI in June, and NIR spectra in Apr and Aug, respectively. TGI, LCI and GLI are the optimal spectral indices for leaf nitrogen detection, which are highly related to leaf chlorophyll content (Hunt et al., 2013; Lima et al., 2021). The anthocyanin reflectance index (ARI) can be used to estimate anthocyanin concentration (Kior et al., 2021). Santini et al. (2021) found four SNPs associated with anthocyanin content in P. halepensis, suggesting that VIs are associated with genomic information. Some VIs, such as GLI in June and GNDVI in September, showed associations with both tree height and crown area (CA), suggesting the possibility of pleiotropy where these VIs simultaneously influence both growth traits within the same month. These results suggest that the detected spectrum and VIs across different months deserve further attention in exploring their potential adaptive role for slash pine.

In discussing the limitations of our study, it is important to acknowledge the lack of a propagation of error analysis in our current manuscript. We recognize the importance of such an analysis in determining the reliability and applicability of our findings. However, several factors prevented us from including a comprehensive error propagation analysis in this study. First, the limitations of our experimental design and data collection process, including sample size limitations and measurement precision, may have affected the feasibility of conducting a robust error propagation analysis. Second, the selected data analysis methods, which rely on model-based estimation and prediction, have inherent limitations with respect to error propagation. Finally, given the scope and objectives of this study, we faced time and resource constraints, as well as limitations in data availability. As a result, a comprehensive analysis of error propagation was beyond the scope of this study.

## 5 Conclusion

With the development of UAV technology, the collection of multispectral or NIR spectra has greatly increased and conversely decreased in cost. In this paper, we use this technology to reinforce the advantages of using the PS approach in Scots pine to estimate the ability of PS used in conifers independent of sequencing or pedigrees. The heritable variation of growth traits in time series was evaluated, temporal growth strongly influenced the genetic variation of growth traits, and the optimal breeding selection time for tree growth traits was suggested. Two types of GS kernels, including GB and GK, showed satisfactory prediction ability based on the tree growth traits at different months using the pedigree and MV instead of genomic markers, indicating that with high-throughput UAV imagery, phenomic selection using multispectral and VIs was possible and reliable. Our study provides insight into the spectral processes reflecting phenotypic differentiation (in our case, tree growth traits) in a time series of UAV technology. Our new PS approach in slash pine bridges the gap between high-dimensional secondary traits (in our study, multispectral imaging) and individual phenotypes.

## Data availability statement

The original contributions presented in the study are included in the article/supplementary material. Further inquiries can be directed to the corresponding author.

## Author contributions

YL designed the study, conducted the experiment and wrote the manuscript. XY, LT, LW, and LX revised the manuscript. JJ supported the data collection and field experiments and supervised the experiments. QL supervised experiments and performed revisions of the manuscript. All authors contributed to the article and approved the submitted version.
